# Identifying the Physiological Traits of Host-Dependent Endophytes in Grapevines, Using Callus as the Host Material

**DOI:** 10.3390/microorganisms13122791

**Published:** 2025-12-08

**Authors:** Yu-Nuo Zhang, Hong-Yan Hu, Yu Li, Shu-Cun Geng, Jing-Xiu Tang, Xiao-Xia Pan, Ming-Zhi Yang

**Affiliations:** 1 School of Ecology and Environmental Sciences and Yunnan Key Laboratory for Plateau Mountain Ecology and Restoration of Degraded Environments, Yunnan University, Kunming 650504, China; zhangyunuo99@163.com (Y.-N.Z.); hucarrot1215@163.com (H.-Y.H.); liyudeyouxiang0420@163.com (Y.L.); gengshucun99@163.com (S.-C.G.); t224547@163.com (J.-X.T.); 2 Institute of International Rivers and Ecological Security, Yunnan University, Kunming 650504, China; 3 Key Laboratory of Chemistry in Ethnic Medicinal Resources, State Ethnic Affairs Commission and Ministry of Education, School of Ethnic Medicine, Yunnan Minzu University, Kunming 650504, China; pan901805@126.com

**Keywords:** callus, microenvironment, host-dependent endophytes (HDEs), cultivation-recalcitrant, physiological trait

## Abstract

In vitro-cultured plant calli are colonized by diverse endophytes. As these endophytes are inherited from the maternal plant and appear to be highly dependent on the eco-niche of the host cells, they have been termed host-dependent endophytes (HDEs). HDEs occupy the most intimate microbial environment of plant cells. Nevertheless, our understanding of HDEs and their microenvironmental effects on host plants remains limited due to their cultivation-recalcitrant nature. In this study, grapevine (*Vitis vinifera* L. × *V. labrusca* L.) callus was subjected to long-term cultivation in media containing different antibiotics (Q: penicillin; L: streptomycin; Z: nystatin) with the intention of creating grapevine calli with different HDEs. The treated calli were then transferred to an antibiotic-free medium for continuous cultivation. After three cycles of subculture over a total period of 45 days, the endophytic microbiota of the grapevine calli were profiled and their physiological parameters were analyzed. Our results revealed that antibiotic treatments can effectively shape HDEs and create distinct bacterial and fungal HDE microbiota in grapevine calli. Compared to treatment without antibiotics (CK), the Q-treated callus contained more Gram-positive bacterial HDEs but fewer Gram-negative and stress-resistant bacterial HDEs, whereas the Z-treated callus had fewer Gram-positive bacterial HDEs and more Gram-negative, stress-resistant and potentially pathogenic bacterial HDEs. More importantly, grapevine calli with different HDE communities showed varying physiological traits such as respiratory rate, peroxidase activity and total sugar content. Correlative analyses further revealed the functional associations between HDE taxa and callus traits. This work provides an example for studying and utilizing plant HDEs.

## 1. Introduction

The term ‘endophyte’ has been defined as simply referring to microorganisms that inhabit the interior environment of plants during all or part of their life cycle, irrespective of their function [1]. As natural components of plant microecosystems, endophytes benefit or harm host plants in various ways [2], and some endophytes that provide beneficial functions for their hosts have been developed into bio-regents and applied in sustainable agricultural practices [3].

Endophytes are categorized as culturable endophytes (CE) or cultivation-recalcitrant endophytes (CREs) based on their culturability. Early studies primarily focused on the study and application of CEs isolated from surface-sterilized plant tissues. However, advances in culture-independent techniques, such as fatty acid profiling [4] and DNA sequencing-based metagenomic analysis, have revealed that the majority of plant endophytes are CREs [5]. Endophytes can also be classified according to their relationship with the host plant as facultative (those requiring host symbiosis only during specific life stages) or obligate endophytes (those entirely dependent on the host throughout their life cycle) [6]. Obligate endophytes with CRE characteristics exhibit high host eco-niche specificity [7] and are referred to here as host-dependent endophytes (HDEs). It is evident that HDEs occupy the most intimate microbial environment of plant cells. Due to their dependency on and specificity to their hosts, some HDEs can be vertically transmitted across host generations, potentially serving as a type of heritable plant trait [8,9], making them particularly interesting. However, due to methodological limitations, the taxonomic diversity and function of HDEs in plants remain poorly characterized.

Callus, an unorganized cell mass formed through dedifferentiation of explants in in vitro cultures, retains the totipotency to regenerate into a whole plant [10]. Studies have revealed that in vitro-cultured plant calli are colonized by diverse endophytes [5,11,12]. These endophytes were inherited from the maternal plant, and the majority of the callus-hosted-endophytes appear to be HDEs, making callus an idea material for studying plant HDEs. In this study, we established an approach to study the possible eco-physiological functions of plant HDEs using grapevine (*Vitis vinifera* L. × *V. labrusca* L.) callus cultured over a long period as a host material.

## 2. Materials and Methods

### 2.1. Subculture and Propagation of Grape Callus

Shoot tips were aseptically excised in July 2017 from 5-year-old grapevines (*Vitis vinifera* L. × *V. labrusca* L. cv. Rose Honey) maintained in a germplasm repository (Kunming, China) and utilized as explants for shoot-tip-derived callus (ST-callus) induction. The established callus was maintained via 7 years of subculturing (>40 passages) and pre-propagated on gelatin salt (GS) agar medium [containing 30 g/L sucrose, 7.6 g/L agar, 0.1 mg/L NAA (α-naphthaleneacetic acid), and 0.2 mg/L KT (kinetin), pH adjusted to 5.8] under controlled conditions (25 ± 1 °C; 12/12 light/dark cycles) [12]. Callus materials were harvested upon reaching a minimum fresh weight of 20 g per biological replicate.

### 2.2. Antibiotic Treatment of Grape Callus and Sampling

Filter-sterilized antibiotic stock solutions (10×) were aseptically inserted into autoclaved GS agar medium under laminar flow to prepare four distinct formulations (Table 1). Calli were inoculated onto these antibiotic-supplemented media and incubated at 28 °C under a 12 h photoperiod (light/dark cycle) for 14 days. Following treatment, each callus was transferred to antibiotic-free GS medium. After 45 days of culture, physiological and morphological parameters—including anthocyanin accumulation, friability, chlorophyll retention, dry matter content, browning index, and necrosis severity—were quantitatively assessed, with concurrent growth monitoring. Upon reaching the biomass plateau, calli from each treatment were systematically partitioned into three parts for downstream microbiome profiling (3–5 g) and physio-biochemical assays (10 g) and lineage maintenance (residual ~5 g subcultured on fresh GS medium).

### 2.3. Profiling Endophytic Microbiota in Callus

Once the biomass plateau had been reached following 45 days of subculture on an antibiotic-free medium, callus samples (3–5 g per treatment) were collected, snap-frozen in liquid nitrogen, and stored at −80 °C. Three biological replicates per treatment were subsequently subjected to high-throughput sequencing for microbiome analysis at LC-Bio (Hangzhou, China). Genomic DNA was extracted from the callus samples for endophytic microbiota profiling, followed by computational analyses, as previously described [13]. The plant endophytic fungal microbiome was amplified using the standard ITS primers ITS1–ITS7 (5′-GTGARTCATCGAATCTTTG-3′) and ITS4 (5′-TCCTCCGCTTATTGATATGC-3′) targeting the ITS region [14]. For bacterial endophyte analysis, 16S rRNA gene amplification was performed using the universal primers 515F (5′-GTGYCAGCMGCCGCGGTAA-3′) and 806R (5′-GGACTACHVGGGTWTCTAAT-3′) [15]. The raw sequencing data for bacterial and fungal communities have been deposited in the NCBI Sequence Read Archive (SRA) under BioProject accession numbers PRJNA1050922 (bacteria) and PRJNA1050887 (fungi).

### 2.4. Physiological and Biochemical Analyses of Callus

Malondialdehyde (MDA) content was determined using the thiobarbituric acid (TBA) method [16]. Callus tissue (1 g) was homogenized in a mortar with 2 mL of 10% trichloroacetic acid (TCA) and quartz sand, followed by further grinding with additional 10% TCA. The homogenate was transferred to 10 mL centrifuge tubes and centrifuged at 10,000× *g* for 10 min. The resulting supernatant served as the extract. The extract was mixed with a TBA solution and heated in boiling water for 30 min. After centrifugation, the absorbance of the extract was read at 532 nm, and the values were corrected for nonspecific turbidity by subtracting the absorbance at 600 nm. The concentration of MDA was calculated using its extinction coefficient.

The catalase (CAT) activity was determined according to the method described by Upadhyaya et al. [17]. Briefly, 0.5 g of callus tissue was homogenized in ice-cold 0.05 M Tris-HCl buffer (pH 7.0) containing 0.001 M EDTA and 0.003 M MgCl_2_. The homogenate was centrifuged at 10,000× *g* for 10 min at 4 °C, and the supernatant was collected as the crude enzyme extract. Catalase activity was measured by monitoring the decomposition rate of hydrogen peroxide (H_2_O_2_) at 240 nm. The reaction mixture (3.2 mL final volume) consisted of 3.0 mL of 50 mM phosphate buffer (pH 7.4), 0.2 mL of 3% (*v*/*v*) H_2_O_2_, and a suitably diluted enzyme extract. The decrease in absorbance at 240 nm, corresponding to the reduction of H_2_O_2_, was recorded for 1 min. One unit of enzyme activity (U) was defined as a decrease of 0.01 absorbance units per minute under the assay conditions.

Peroxidase (POD) activity was determined following a modified spectrophotometric method [18]. Fresh callus tissue (1 g) was homogenized in 5 mL of 0.02 M KH_2_PO_4_ solution and centrifuged at 3000 r/min for 10 min. The supernatant was collected as the crude enzyme extract. For the assay, the reaction mixture (final volume 4 mL) contained 3 mL of 50 mM phosphate buffer (pH 6.0), 1 mL of 1% (*v*/*v*) hydrogen peroxide (H_2_O_2_), and 1 mL of 1% (*w*/*v*) guaiacol. A 1 mL aliquot of the enzyme extract was added to initiate the reaction, while a reference cuvette contained 3 mL of the reaction mixture and 1 mL of KH_2_PO_4_ solution instead of the enzyme extract. The increase in absorbance at 470 nm due to the oxidation of guaiacol was monitored for 10 min at 25 °C. One unit of peroxidase activity (U) was defined as an increase in absorbance of 0.01 per minute under the assay conditions, consistent with the CAT activity definition.

To determine the total phenolic content (TP), total flavonoid content (TF), soluble protein content (TPr) and chlorophyll content (Chl), fresh callus tissues (0.1–0.5 g) were homogenized in 1 mL of methanol and diluted to a final volume of 10 mL for spectrophotometric analysis. TP was quantified at 760 nm using the Folin–Ciocalteu method [19], while TF was determined at 510 nm via the aluminum chloride colorimetric assay [20]. TPr was measured at 595 nm with the Bradford method, as modified by Qu [21]. Chl was calculated from absorbance values at 665 nm and 649 nm according to the protocol established by Scrob et al. [22]. All measurements were performed in triplicate using a U-5100 UV/VIS spectrophotometer (Shanghai, China). Calibration curves for TP, TF, TPr, and Chl were established with gallic acid, quercetin, bovine serum albumin, and purified chlorophyll as the respective standards. Appropriate solvent blanks were used throughout to ensure analytical accuracy.

The size and viability of callus cells were determined according to the method described by Strober, with modifications [23]. For cell size measurement, 1 g of callus tissue was directly suspended in 1 mL of distilled water and gently agitated to disperse the cells, omitting the centrifugation step. A 10 μL aliquot of the suspension was rapidly transferred to a glass slide using a micropipette, and the diameters of 100 randomly selected cells were measured under a light microscope (×400 magnification). For cell viability assessment, the staining protocol was simplified by mixing 100 μL of the cell suspension with an equal volume of 0.4% (*w*/*v*) trypan blue solution in a microcentrifuge tube. After 3 min of incubation at room temperature, a 10 μL aliquot was placed on a slide, and the viability ratio was calculated by counting the proportion of unstained (viable) versus stained (nonviable) cells among 100 cells observed microscopically.

All the above experiments were repeated for three or more biological replicates to ensure the reproducibility of results.

### 2.5. Statistical Analysis of Data

Bioinformatic analysis was performed using the OmicStudio tools at https://www.omicstudio.cn/tool (20 August 2025). Alpha diversity indices were analyzed to assess microbial diversity across sample groups. Significant intergroup differences were statistically validated using Kruskal–Wallis tests supplemented by Wilcoxon rank-sum tests. Principal coordinate analysis (PCoA) was performed on amplicon sequence variants (ASVs) detected in callus tissues subjected to distinct antibiotic treatments to evaluate beta diversity at the ASV level.

Physiobiochemical and morphological parameters were examined through one-way ANOVA with Tukey’s multiple range test to determine significance thresholds (*p* < 0.05), which are presented in box plot format. Spearman’s rank correlation analysis elucidated associations between the endophytic microbiota and host physiological indicators, with key correlations (|ρ| > 0.6, *p* < 0.01) between critical ASVs and metrics, including peroxidase activity and total protein content, visualized through correlation network diagrams. Functional predictions for fungal and bacterial taxa were made using FUNGuild (https://www.bioincloud.tech, 20 August 2025) and BugBase (https://bugbase.cs.umn.edu/, 20 August 2025), respectively [24].

For the majority of statistical analyses and graphical outputs, R packages (https://www.omicstudio.cn/home, 20 August 2025) were used, complemented by Microsoft Excel (version 2019) and Microsoft PowerPoint (version 2019) software suites.

## 3. Results

### 3.1. Antibiotic Treatments Conferred Effects Shaping the Endophytic Microbiota in Grapevine Callus

**Figure A1:**
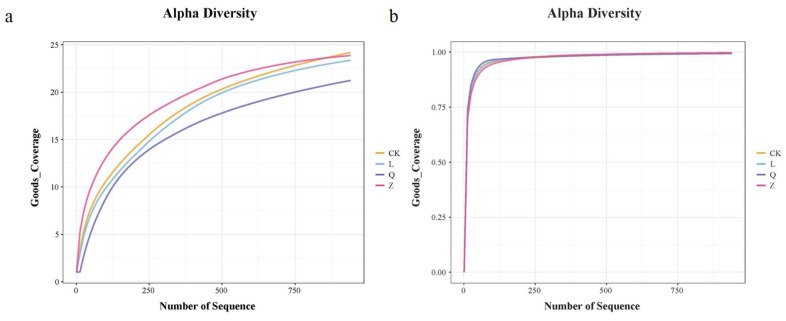
The Goods coverage rarefaction curves for 16r rRNA (**a**) and ITS DNA (**b**) sequencing in grapevine callus samples of different treatments.

Profiling the bacterial endophytes from 36 callus samples resulted in 1,129,350 raw 16S rDNA sequences, with an average Q30 score of 94%. After read splicing and filtering, a total of 4,230,732 high-quality sequences (clean tags) remained (Table A1). These were assigned to 311 bacterial amplicon sequence variants (ASVs), 130 genera, and 11 phyla (Table A2). Similarly, fungal endophyte profiling yielded 1,005,227 raw ITS reads, with an average Q30 score of 96%. After filtering low-quality data, chimeric sequences, and plant-origin reads, 955,713 high-quality ITS sequences obtained, which were assigned to 252 fungal ASVs, 94 genera, and 5 phyla (Table A3 and Table A4). Rarefaction analyses confirmed that sequencing depth was adequate to capture the full microbial diversity present in the samples (Figure A1).

The alpha diversity of endophytes (both bacterial and fungal) in grapevine callus varied greatly between replicates of the same sample, whereas differences in alpha diversity between groups of samples from different treatments were not statistically significant (Table 2 and Table 3).

**Figure 1:**
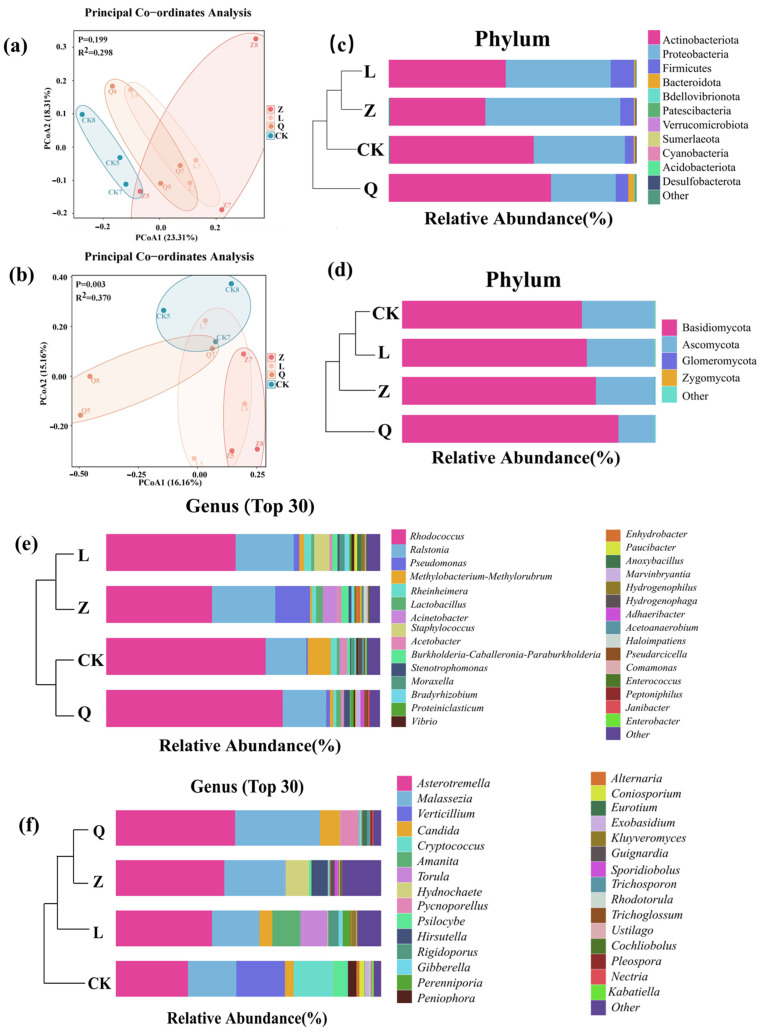
Impact of antibiotic treatments on beta diversity and composition of endophytic microbiota in grapevine calli. (**a**,**b**) Principal coordinate analysis (PCoA) based on bacterial (**a**) and fungal (**b**) endophytes; (**c**,**d**) compositional and clustering analyses of most abundant bacterial (**c**) and fungal (**d**) phyla; (**e**,**f**) compositional and clustering analyses of 30 most abundant bacterial (**e**) and fungal (**f**) endophytes at genus level.

In the principal coordinate analysis (PCoA), both fungal and bacterial endophytic microbiota of the antibiotic-treated groups of calli were clearly separate from the control group, demonstrating the effective alteration of endophytic microbiota in the callus tissue by antibiotics. The fungal communities exhibited statistically significant treatment-driven reshaping (*p* = 0.003; R^2^ = 0.370), indicating selective antibiotic pressures across groups. In contrast, the bacterial communities showed non-overlapping 95% confidence ellipses between the control group (CK-callus) and antibiotic-treated groups (Z/L/Q-callus) (*p* = 0.199; R^2^ = 0.298). Notably, higher intra-group heterogeneity within replicates of Z-treated callus samples was observed (Figure 1a,b).

The predominant bacterial endophytes detected in the grapevine calli included four phyla, namely Actinobacteriota, Proteobacteria, Firmicutes and Bacteroidota, accounting for more than 99% (relative abundance) of the total detected endophytic bacteria, but slight differences of the relative abundances (RAs) of different phylum in each sample groups were observed. Actinobacteriota was the most dominant bacterial phylum in all detected grapevine calli, with RAs ranging from 39% in Z-callus to 65% in Q-callus (Figure 1c, Table A5). Notably, Proteobacteria was the most dominant bacterial phylum in Z-callus specifically, with an RA of 54.7%; Firmicutes demonstrated preferential enrichment in L-callus (9.4% RA). Among all treatments, Q-callus exhibited the highest diversity (9 phyla), contrasting with the CK and L-callus (6 phyla each). Accordingly, fungal endophytes were dominated by the phylum Basidiomycota (RA = 77.43%), followed by the phylum Ascomycota (RA = 22.51%) (Figure 1d, Table A6). Compared to CK-callus, Q-callus obviously promoted the RA of the fungal phylum Basidiomycota while suppressing the phylum Ascomycota in grapevine callus (Figure 1e).

Significant impacts of antibiotic treatment on endophytic genera in grapevine callus were observed in this experiment (Figure 1, Table A7 and Table A8). CK-callus was dominated by the bacterial genera *Rhodococcus*, *Ralstonia*, *Methylobacterium-Methylorubrum*, *Rheinheimera*, and *Acetobacter*. Meanwhile, Z-callus showed primary colonization by *Rhodococcus*, *Ralstonia*, *Pseudomonas*, *Acinetobacter*, and *Burkholderia-Caballeronia-Paraburkholderia*. L-callus exhibited predominance of *Rhodococcus*, *Ralstonia*, *Staphylococcus*, *Rheinheimera*, and *Pseudomonas*; whereas Q-callus featured *Rhodococcus*, *Ralstonia*, *Stenotrophomonas*, *Lactobacillus*, and *Marvinbryantia* as core taxa (Figure 1e). Fungal communities in CK-callus were characterized by a predominance of *Asterotremella*, *Verticillium*, *Malassezia*, *Cryptococcus*, and *Psilocybe*. Comparatively, Z-callus hosted *Asterotremella*, *Malassezia*, *Hydnochaete*, unclassified *Elsinoaceae*, and *Hirsutella* as major components. L-callus assemblages presented predominance of *Asterotremella*, *Malassezia*, *Amanita*, *Torula*, and *Candida*, while Q-callus displayed a prevalence of *Asterotremella*, *Malassezia*, *Candida*, *Pycnoporellus*, and *Eurotium* (Figure 1f).

**Figure 2:**
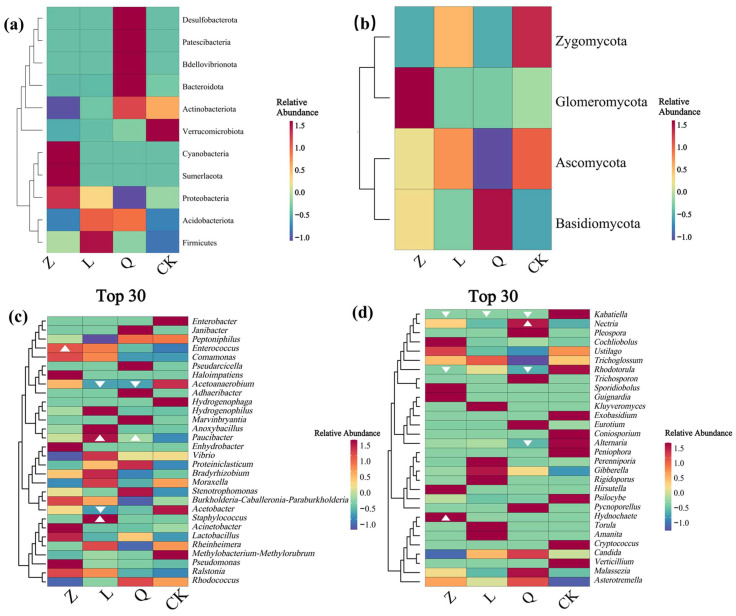
The different distributions of endophytic phyla and genera in grapevine calli driven by the antibiotic treatments. (**a**,**b**) Phylum-level distribution of bacterial (**a**) and fungal (**b**) endophytes; (**c**,**d**) genus-level profiles of bacterial (**c**) and fungal (**d**) endophytes. White triangles indicate taxa with significant differences (*p* < 0.05) in relative abundance: ▲ increase; ▼ decrease.

Difference analysis of the bacterial endophytes revealed that Verrucomicrobiota was enriched in CK-callus, whereas nystatin treatment (Z-callus) led to an increase in the relative abundances of Sumnerlaeota and Cyanobacteria. Streptomycin treatment (L-callus) resulted in a higher abundance of Firmicutes, and penicillin treatment (Q-callus) elevated the levels of Bacteroidota, Bdellovibrionota, Patescibacteria, and Desulfobacterota (Figure 2a). Fungal HDEs showed Zygomycota enrichment in CK-callus, Glomeromycota dominance in Z-callus, and Basidiomycota accumulation in Q-callus (Figure 2b).

At the genus level, nystatin treatment led to a substantial enrichment of the genera *Enterococcus* (12.72%) and *Acinetobacter* (5.25%) within the bacterial communities (Figure 2c) while simultaneously elevating the fungal genus *Hydnochaete* (8.78%) to a dominant position (Figure 2d). Exposure to streptomycin resulted in significant increases in the bacterial genera *Staphylococcus* (5.60%) and *Paucibacter* (1.07%) (Figure 2c) but reduced the abundance of the fungal taxa *Kabatiella* (0%) and *Chaetomium* (0%) (Figure 2d). Penicillin treatment selectively enriched the bacterial genus *Paucibacter* (0.29%) while promoting the fungal genera *Nectria* (0.42%) and *Meyerozyma* (0.19%) (Figure 2c,d). Notably, *Colidextribacter* exhibited consistent depletion across all antibiotic regimens, indicating high antimicrobial susceptibility. In contrast, *Paucibacter* showed synchronized enrichment under both ampicillin and streptomycin treatments, suggesting potential antibiotic tolerance. Together, these taxon-specific shifts in abundance illustrate how antibiotic interventions reshape the endophytic architecture of grape callus through selective suppression and enrichment.

### 3.2. The Predicted Functions of the Endophytic Microbiota in Grapevine Callus Were Changed Due to Antibiotic Treatment

**Figure 3:**
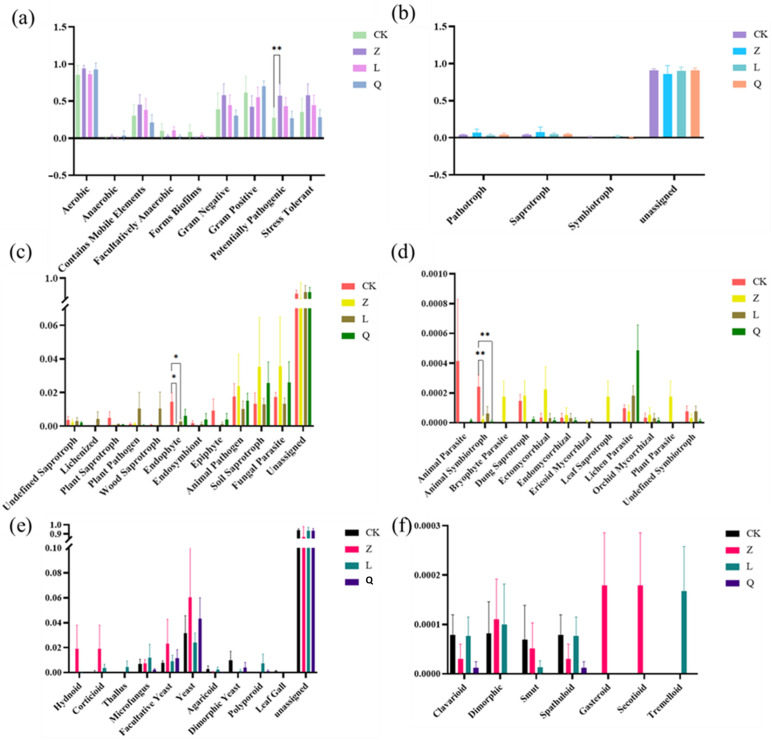
Functional prediction of endophytes in differently treated grapevine calli. (**a**) Phenotype prediction (Bugbase) of bacterial endophytes in differently treated grapevine calli. Functional prediction of the fungal endophytes in differently treated grapevine calli was performed using (**b**) the abundance of fungal trophic mode, (**c**,**d**) guild, and (**e**,**f**) growth form, as predicted by the FUNGuild program. “*” indicates a significant difference (** *p* < 0.01; * *p* < 0.05).

Phenotype prediction by BugBase analysis revealed that L-callus was predicted to preferentially enrich Gram-negative bacteria (e.g., Proteobacteria) while also increasing the relative abundance of bacteria predicted to be potentially pathogenic and containing mobile elements. In contrast, penicillin predominantly affected Gram-positive populations (e.g., Actinobacteriota) and was associated with a reduction in predicted biofilm-forming endophytic bacteria in Q-callus. Z-callus showed an increased abundance of predicted and strictly anaerobic and stress-tolerant taxa (Figure 3a), which could potentially influence the stability of the plant microenvironment. Although the observed shifts did not reach statistical significance, the increased prevalence of predicted mobile elements and potentially pathogenic bacteria in Z-callus and L-callus suggests a potential long-term implication for host health, based on these computational predictions. Furthermore, the predicted reduction in biofilm-forming ability observed in all antibiotic-treated calli may indicate potential impairment of bacterial colonization efficiency in plant tissues.

The functional potential of the fungal communities across different callus treatments was inferred using the FUNGuild annotation system (Figure 3b–f). Trophic mode analysis revealed that the fungal community was dominated by the predicted saprotrophic fungi, followed by symbiotic and pathogenic trophic types. Nystatin treatment was associated with an increase in the relative abundances of predicted saprotrophic and pathogenic fungi, while the proportion of symbiotic fungi in the grapevine callus was reduced. In contrast, streptomycin exposure elevated symbiotic fungal abundance, though no significant differences were observed across treatments (Figure 3b). Guild abundance analysis indicated significant reductions in animal symbiotroph abundance in Z-callus (*p* = 0.008) and Q-callus (*p* = 0.0013), while endophytic taxa were markedly depleted in both Z-callus (*p* = 0.023) and L-callus (*p* = 0.046) (Figure 3c,d). Regarding predicted growth forms, in Z-callus, yeast-associated morphologies (yeast, facultative yeast) and complex sporocarp structures (corticioid, hydnoid, gasteroid, secotioid) were enriched, while streptomycin treatment favored microfungus, polyporoid, thallus, and tremelloid morphologies. Q-callus showed a reduction in most predicted fungal growth forms (Figure 3e,f).

**Figure 4:**
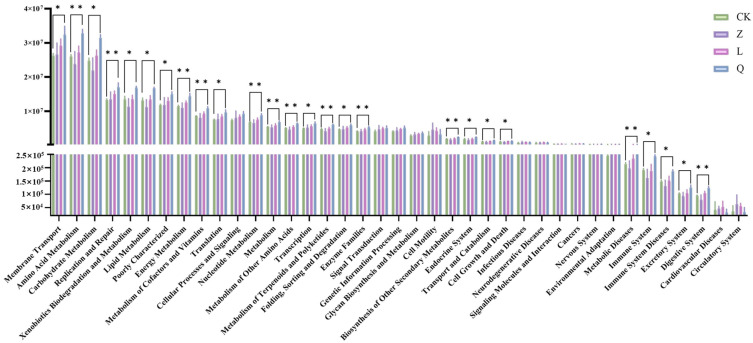
Function prediction of bacterial endophytes associated to KEGG pathways in differently treated grapevine calli. “*” indicates a significant difference between the two (** *p* < 0.01; * *p* < 0.05).

Bacterial functional prediction using PICRUSt2 revealed significant differences in the functional composition of endophytic bacteria in grape callus under different antibiotic treatments. The bacterial endophytes were associated to 39 secondary metabolic pathways, among which 24 pathways were significantly altered after antibiotic exposure (*p* < 0.05, Figure 4). Notably, membrane transport, amino acid metabolism, and carbohydrate metabolism were among the most abundant functional categories. Compared to the CK-callus, both Q-callus and L-callus showed significant upregulation of most secondary metabolic pathways related to metabolism. In contrast, Z-callus did not show significant upregulation in pathways such as membrane transport; instead, the treatment led to pronounced downregulation of the majority of metabolic pathways. These results underscore the antibiotic-specific reshaping of metabolic potential in endophytic bacteria associated with grape callus.

### 3.3. The Grapvine Callus with Different Endophytic Microbiota Exhibited Differential Physio-Ecological Characteristics

**Figure 5:**
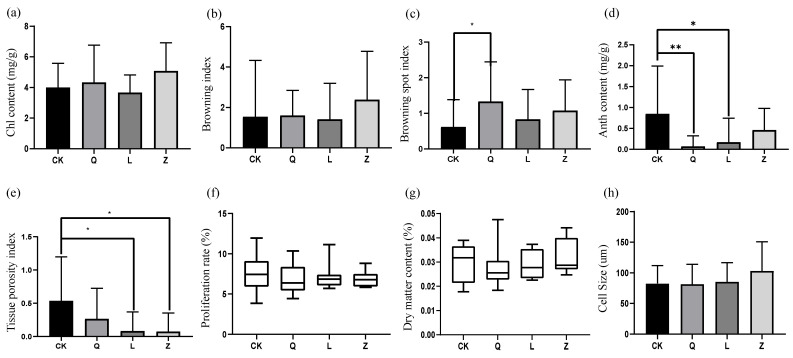
Morphophysiological impacts of antibiotic treatment on grapevine calli. (**a**) Chlorophyll (Chl) content, (**b**) browning index, (**c**) browning spot index, (**d**) anthocyanin (Anth) content, (**e**) tissue porosity, (**f**) proliferation rate, (**g**) dry matter content, and (**h**) cell diameter (** *p* < 0.01; * *p* < 0.05).

Antibiotic treatments induced distinct morphological and physiological alterations in grape callus (Figure 5). While penicillin and nystatin intensified tissue Chl content, streptomycin reduced it without statistical significance (Figure 5a). Penicillin and nystatin intensified browning, while streptomycin inhibited browning; however, there was not a significant difference (Figure 5b). A significant increase in the browning spot index was observed in Q-callus (Figure 5c). Secondary metabolism was notably impaired, with universal suppression of anthocyanin biosynthesis—penicillin showed highly significant inhibition (*p* < 0.01), and streptomycin showed moderate suppression (*p* < 0.05) (Figure 5d). Histomorphological quantification confirmed significant reductions in tissue porosity, particularly under the streptomycin and nystatin treatments (*p* < 0.05) (Figure 5e). The proliferation rate and dry matter content did not differ significantly across the treatment groups. (Figure 5f,g). Assessment of cell morphology showed an upward trend in cell volume, which was the greatest in Z-callus; however, statistical analysis did not detect any significant differences between the treatment groups (Figure 5h).

**Figure 6:**
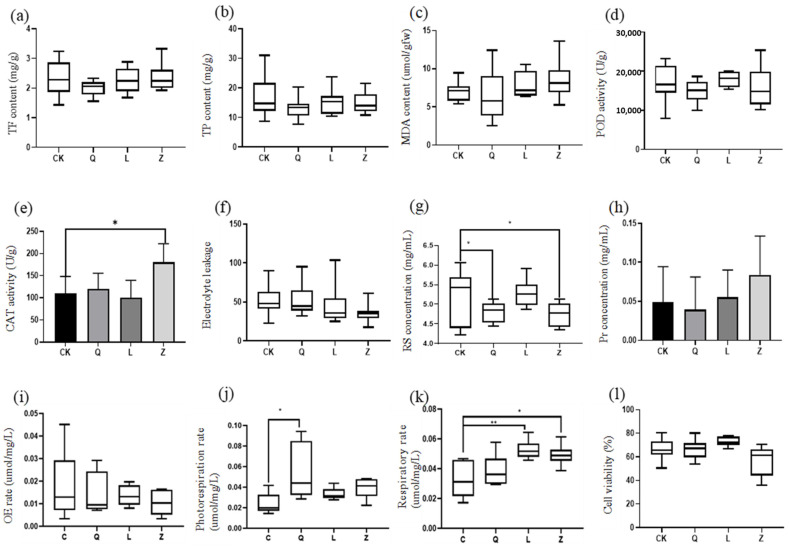
Physiological and biochemical responses in antibiotic-treated grapevine calli. (**a**) total flavonoid (TF) content, (**b**) total phenol (TP) content, (**c**) malondialdehyde (MDA) content, (**d**) peroxidase (POD) activity, (**e**) catalase (CAT) activity, (**f**) electrolyte leakage, (**g**) reducing sugar (RS) concentration, (**h**) protein (Pr) concentration, (**i**) oxygen evolution (OE) rate, (**j**) photorespiration rate, (**k**) respiratory rate, and (**l**) cell viability (** *p* < 0.01; * *p* < 0.05).

Integrated physiological profiling revealed multidimensional regulatory effects of antibiotics on stress adaptation mechanisms in callus. Penicillin treatment reduced the total flavonoid content, whereas streptomycin and nystatin induced non-significant upward trends in grapevine calli (Figure 6a). A parallel response pattern was observed for total phenolic compounds across treatments (Figure 6b). Membrane stability assessments demonstrated a 17.4% reduction in malondialdehyde (MDA) levels under penicillin treatment, contrasted by non-significant elevations in L-callus and Z-callus (Figure 6c). Antioxidant enzyme systems exhibited differential modulation: peroxidase (POD) activity decreased in Q-callus and Z-callus but increased in L-callus (non-significant), while catalase (CAT) activity was significantly enhanced in Z-callus (*p* < 0.05), following the hierarchy L, CK and Q-callus (Figure 6d,e). Cell membrane integrity assays confirmed nystatin-induced reduction in relative electrolyte leakage (*p* < 0.01), with L-callus and Q-callus showing intermediate effects, indicating reinforced membrane stability (Figure 6f).

Growth-related metabolic analyses demonstrated antibiotic-specific impacts: all treatments significantly decreased reducing sugar concentrations, particularly in Q-callus and Z-callus (*p* < 0.01) (Figure 6g). Total protein content was markedly reduced in Q-callus (*p* < 0.05) but elevated in L-callus, reaching peak levels in Z-callus (Figure 6h). Energy metabolism profiling revealed generalized respiratory rate enhancement, with L-callus and Z-callus showing highly significant (*p* < 0.01) and significant (*p* < 0.05) increases, respectively. Photorespiration rates increased across treatments, though penicillin-induced elevation exhibited significant inter-replicate variability. Photosynthetic oxygen evolution rates showed treatment-dependent variations without statistical significance (Figure 6i–k). Cellular viability assays highlighted penicillin- and streptomycin-mediated enhancement versus nystatin-induced suppression, ranked as L > Q > C > Z (*p* < 0.05) (Figure 6l). These findings collectively elucidate the compound-specific reprogramming of metabolic networks governing stress tolerance, energy flux, and cellular homeostasis in plant callus systems.

### 3.4. Associations of Endophytic Taxa with the Callus Physio-Ecological Traits

**Figure 7:**
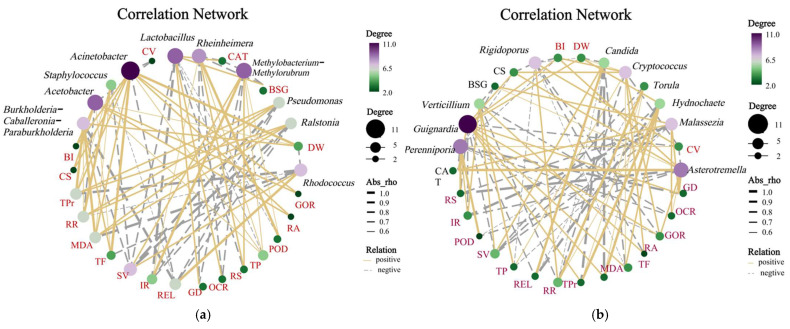
Co-occurrence networks of the top 10 most abundant (**a**) bacterial or (**b**) fungal genera in grapevine calli versus morphophysiological parameters.

Correlation analysis revealed associations between dominant endophytes and callus physiological traits (Figure 7). Among bacterial genera, *Pseudomonas* was positively correlated with browning index and dry weight and demonstrated significant links to POD and CAT enzymatic activities. *Pelomonas* exhibited broad synergistic relationships with total flavonoids, phenolics, browning spots, and cellular viability. *Rhodococcus* was specifically associated with dry weight, contrasting with *Methylobacterium*, which showed inverse correlations with photosynthetic and photorespiratory oxygen flux (all *p* < 0.05 unless specified) (Figure 7a).

For fungal taxa, *Cryptococcus* was negatively correlated with browning spots, whereas *Hydnochaete* was positively linked to cellular viability. Three fungal genera (*Asterotremella*, *Malassezia*, and *Hydnochaete*) demonstrated significant associations with total protein content. Two genera (*Asterotremella* and *Hydnochaete*) were correlated with reducing sugars, while three others (*Saccharomyces*, *Verrucariaceae_unclassified*, and *Rigidoporus*) showed positive correlations with electrolyte leakage (*p* < 0.05) (Figure 7b).

## 4. Discussion

HDEs identified in long-term subcultured grape callus exhibited substantial structural divergence from the endophytic microbiota of field-grown grape plants. Natural grapevine microbiota are typically dominated by *Proteobacteria* and *Firmicutes*, enriched with functional taxa associated with plant disease resistance and nutrient assimilation, such as *Burkholderia* and *Bacillus* [1]. In contrast, the callus-associated communities were primarily composed of Actinobacteriota (52.80%) and Basidiomycota (82.59%), with *Rhodococcus* (52.21%) and *Asterotremella* (37.39%) emerging as the dominant genera. This disparity likely arises from the unique selective pressures of in vitro culture conditions, where stable carbon sources (e.g., sucrose) and phytohormones (e.g., NAA, KT) may enrich microbiota adapted to high-osmolarity environments and biosynthetic demands [5]. Additionally, the absence of vascular systems and environmental stress signaling in callus tissues may favor the stable colonization of obligate symbionts or metabolically dependent endophytes. Notably, the abundance of *Rhodococcus* showed a significant positive correlation with callus dry weight ratio (Figure 7), suggesting its potential role in modulating host carbon metabolism to maintain cellular homeostasis. These findings underscore how in vitro culture conditions reshape endophytic assemblies, favoring taxa with specialized metabolic adaptations over those optimized for ecological interactions in natural plant systems.

Compared to previous studies on endophytic microbiota in *Vitis vinifera* L. × *V. labrusca* L. callus [12], the bacterial genera *Ralstonia* and *Pseudomonas* remained dominant in this study, consistent with their roles in nutrient acquisition and stress tolerance. Notably, *Methylobacterium-Methylorubrum* and *Rhodococcus* emerged as unique keystone taxa in the current system, potentially linked to methanol metabolism and lipid degradation, respectively, suggesting adaptive responses to carbon source shifts or host-derived metabolites during long-term subculture. In contrast, *Acinetobacter* and *Halomonas*, previously reported as dominant taxa [12], were undetected here, possibly due to differences in subculture frequency or medium salinity. Xiang et al. identified *Erysiphe* and *Sarocladium* as predominant fungal genera in Rose Honey tissue-cultured seedlings, alongside bacterial enrichment of *Cupriavidus* and *Cellvibrio* [13], which may be associated with phototrophic metabolism, cell wall remodeling, and nutrient cycling. By comparison, the current callus system was dominated by *Rhodococcus* and *Methylobacterium* in bacteria and *Astrotremella* and *Verticillium* in fungi, implying adaptations to phenolic degradation, carbon utilization, and oxidative stress. The common presence of *Ralstonia* across studies likely reflects its broad adaptability to plant endophytic niches. These disparities underscore the integrated effects of tissue type (callus versus seedlings), physiological state, and subculture-induced selection pressures on endophytic community assembly.

Among the dominant genera identified, *Pseudomonas* and *Methylobacterium* exhibited functionally distinct roles. The abundance of *Pseudomonas* showed significant positive correlations with host peroxidase (POD, r = 0.68) and catalase (CAT, r = 0.72) activities (*p* < 0.05), suggesting its potential role in alleviating oxidative stress by enhancing antioxidant defenses, thereby stabilizing callus cell viability [25]. *Methylobacterium*, previously implicated in plant nitrogen metabolism via bacterial urease activity [26,27,28]), displayed a significant negative correlation with photosynthetic and respiratory rates in this study (r = −0.61, *p* < 0.05), implying its involvement in modulating carbon metabolism to influence host energy allocation. Notably, while *Rhodococcus*, *Methylobacterium-Methylorubrum*, *Rheinheimera*, and *Staphylococcus* reduced callus browning indices, these effects did not reach statistical significance, contrasting with prior findings where *Bacillus* sp. strain ST-B1. A, isolated by Wang et al., exacerbated browning in grape callus. Similarly, M. Pirttilä et al. reported delayed tissue browning in *Pinus sylvestris* callus inoculated with *M. extorquens*, *P. synxantha*, and *R. minuta* [29]. Fungal HDEs, particularly *Asterotremella*, demonstrated significant positive correlations with total protein content (r = 0.54) and reducing sugar concentrations (r = 0.49, *p* < 0.05), potentially mediated by extracellular enzyme secretion to enhance host carbon utilization from the medium. However, the molecular mechanisms underlying these taxa–host interactions, especially direct metabolic crosstalk, require further validation.

This study establishes grapevine callus as a robust model system for investigating functional relationships between HDEs and plant physiology through antibiotic-mediated community modulation. Antibiotic-specific effects were observed in HDE assembly, with penicillin selectively enriching Gram-positive taxa (e.g., *Paucibacter*) while nystatin promoted Gram-negative bacteria and suppressed *Colidextribacter*, demonstrating precision microbial manipulation. Taxon–host phenotype correlations revealed associations of *Rheinheimer* with phenolic compounds and cellular viability, *Pseudomonas* with elevated antioxidant enzymes (POD, CAT), and *Rhodococcus* with biomass accumulation. The fungal endophytes *Astrotremella*, *Malassezia*, and *Hydnochaete* showed positive correlations with total protein content. The persistence of core taxa (*Rhodococcus*, *Astrotremella*) validated the callus system’s reliability for studying obligate endophytes. By developing an “antibiotic remodeling–multiomics profiling–functional validation” framework, this research pioneers a new approach to investigating HDEs, enabling future studies on synthetic community reconstruction and molecular interaction mechanisms for crop stress resilience and propagation optimization. These findings position HDEs as dynamic regulators of host physiology through community composition, providing methodological foundations for agricultural microbiome innovation.

However, functional identification of HDEs using callus tissue present significant challenges when investigating non-core HDE taxa. The spatial heterogeneity of these HDEs within callus tissue leads to random distribution across subcultured samples. This may also account for the substantial variation in alpha diversity observed within treatments.

## Figures and Tables

**Table 1:** Description of antibiotic formulations tested.

Treatments	Antibiotic Concentrations and Stock Solution Types	Rationale for Antibiotic Selection	Expected Activity Spectrum
CK: None (Control)	-	Serves as a baseline to assess the natural state of the endophytic community and physiological parameters without any antibiotic perturbation.	Natural, unmodulated host-dependent endophyte (HDE) community.
Q: Ampicillin	260 mg/L, sterile aqueous solution	Selected for its primary activity against Gram-positive bacteria by inhibiting cell wall synthesis. Aims to selectively perturb this segment of the bacterial HDE community.	Reduction in Gram-positive bacteria (e.g., Actinobacteriota, Firmicutes); potential enrichment of Gram-negative bacteria and fungi due to reduced competition.
L: Streptomycin	100 mg/L, sterile aqueous solution	Chosen for its broad-spectrum activity against Gram-negative bacteria and some Gram-positive bacteria by inhibiting protein synthesis. Aims to broadly reduce bacterial load.	Significant reduction in overall bacterial abundance; potential competitive release for fungi within the HDE community.
Z: Nystatin	50 mg/L, DMSO solution	Used for its specific antifungal activity by binding to ergosterol in fungal membranes. Aims to selectively target and reduce the fungal component of the HDEs.	Significant suppression of fungal endophytes; potential shift in bacterial community structure due to altered bacterial–fungal interactions.

**Table 2:** Alpha diversities of bacterial endophytes in grapevine calli under different treatments.

Samples	Observed OTUs	Shannon	Simpson	Chao1	Pieloue	Ace
Z-callus	50.3 ± 26.7 a	2.9 ± 1.3 a	0.7 ± 0.2 a	50.3 ± 26.6 a	0.5 ± 0.2 a	50.3 ± 26.6 a
L-callus	54.3 ± 11.0 a	2.7 ± 0.6 a	0.7 ± 0.1 a	54.5 ± 10.8 a	0.5 ± 0.1 a	54.9 ± 10.8 a
Q-callus	54.7 ± 15.6 a	2.0 ± 0.5 a	0.5 ± 0.1 a	54.8 ± 15.6 a	0.4 ± 0.1 a	55.2 ± 15.8 a
CK-callus	67.3 ± 16.0 a	2.2 ± 0.6 a	0.6 ± 0.2 a	67.5 ± 9.3 a	0.4 ± 0.1 a	67.9 ± 9.4 a

Note: Different lowercase letters within the same column indicate significant differences (*p* < 0.05); the same notation applies to subsequent figures and tables.

**Table 3:** Alpha diversities of fungal endophytes in grapevine calli under different treatments.

Groups	Observed_Species	Shannon	Simpson	Chao1	Pielou_e
Z	24.0 ± 13.5 a	2.7 ± 0.9 a	0.8 ± 0.1 a	25.1 ± 12.1 a	0.6 ± 0.1 a
L	25.3 ± 6.5 a	2.4 ± 0.6 a	0.7 ± 0.1 a	28.7 ± 5.7 a	0.5 ± 0.1 a
Q	22.3 ± 2.5 a	2.2 ± 0.7 a	0.7 ± 0.2 a	25.9 ± 5.5 a	0.5 ± 0.2 a
CK	24.3 ± 4.5 a	2.2 ± 0.4 a	0.7 ± 0.0 a	32.9 ± 4.5 a	0.5 ± 0.1 a

## Data Availability

All plant lines and microbial strains are available upon request. All data and materials are available in the manuscript and in the NCBI under the accession numbers PRJNA1050887 (fungi, https://submit.ncbi.nlm.nih.gov/subs/bioproject/SUB14031423/overview, 20 August 2025) and PRJNA1050922 (bacteria, https://submit.ncbi.nlm.nih.gov/subs/bioproject/SUB14032487/overview, 20 August 2025). All study data are included in the article.

## Abbreviations

The following abbreviations are used in this manuscript:

HDEs	Host-dependent endophytes
CE	Culturable endophyte
CRE	Cultivation-recalcitrant endophyte
GS	Gelatin salt
TCA	Trichloroacetic acid
TBA	Thiobarbituric acid
MDA	Malondialdehyde
CAT	Catalase
POD	Peroxidase
TP	Phenolic content
TF	Total flavonoid content
TPr	Soluble protein content
Chl	Chlorophyll content
PCoA	Principal coordinate analysis

## Appendix A

**Table A1:** Statistics of the 16S rRNA amplicon sequences in callus samples treated with different antibiotics.

Sample	Raw Tags	Raw Bases	Valid Tags	Valid Bases	Valid (%)	Q20 (%)	Q30 (%)	GC (%)
Z1	96,053	48.03 M	90,734	37.61 M	94.46	98.41	94.78	54.75
Z2	80,313	40.16 M	74,887	31.48 M	93.24	97.18	91.93	52.54
Z3	85,226	42.61 M	81,397	33.66 M	95.51	98.45	94.61	55.24
L1	96,357	48.18 M	87,917	36.48 M	91.24	98.36	94.69	54.75
L2	86,562	53.28 M	81,501	41.83 M	95.25	98.25	94.14	55.22
L3	83,848	51.92 M	98,914	41.06 M	95.25	98.21	94.08	54.83
Q1	93,823	46.91 M	87,355	35.85 M	93.11	98.48	94.93	55.57
Q2	84,463	42.23 M	80,661	33.16 M	95.5	98.08	93.9	55.77
Q3	114,824	57.41 M	18,086	45.45 M	95.87	98.11	93.88	54.86
CK1	88,249	44.12 M	83,709	34.47 M	94.86	98.51	95.03	55.45
CK2	85,577	42.79 M	81,794	33.79 M	95.58	98.19	94.21	55.18
CK3	94,055	47.03 M	88,988	36.34 M	94.61	98.47	94.79	55.79

**Table A2:** Statistics of the identified endophytic bacteria at different taxonomic levels in callus samples.

Samples	Reads	ASVs	Species	Genera	Families	Orders	Classes	Phyla
Z1	87,375	64	53	45	34	23	11	6
Z2	68,585	73	55	43	34	25	8	6
Z3	78,244	22	19	19	17	13	8	5
L1	83,990	71	58	47	36	27	11	6
L2	97,196	60	44	39	30	20	8	6
L3	94,436	50	43	41	34	22	11	6
Q1	83,850	74	64	56	44	29	13	8
Q2	76,520	43	33	30	25	17	9	7
Q3	84,380	55	43	42	32	22	8	7
CK1	80,725	78	62	52	35	24	8	6
CK2	78,135	59	52	47	37	22	11	7
CK3	85,223	72	50	48	33	22	11	6

**Table A3:** Statistics of the ITS amplicon sequences in callus samples treated with different antibiotics.

Sample	Raw Tags	Raw Bases	Valid Tags	Valid Bases	Valid (%)	Q20 (%)	Q30 (%)	GC (%)
Z1	89,020	44.51 M	85,262	26.59 M	95.78	99.16	97.32	71.44
Z2	82,731	41.37 M	79,209	24.66 M	95.74	99.32	97.45	71.41
Z3	83,564	41.78 M	80,070	24.84 M	95.82	99.24	97.12	66.82
L1	85,221	42.61 M	81,696	25.20 M	95.86	99.02	96.48	70.02
L2	82,173	41.09 M	77,811	24.60 M	94.69	98.49	94.66	72.23
L3	85,340	42.67 M	79,758	25.29 M	93.46	97.84	93.33	72.2
Q1	85,298	42.65 M	81,869	25.8 M	95.98	99.37	97.57	69.95
Q2	86,235	43.12 M	82,403	26.17 M	95.56	99.35	97.62	72.34
Q3	8804	40.50 M	76,739	24.11 M	94.73	98.4	94.38	71.59
CK1	82,093	41.05 M	78,756	24.81 M	95.94	99.39	97.89	71.51
CK2	82,336	41.17 M	77,373	24.41 M	93.97	97.67	93.19	72.39
CK3	81,212	40.61 M	74,767	23.48 M	92.06	99.06	96.58	70.91

**Table A4:** Statistics of the identified endophytic fungi at different taxonomic levels in callus samples.

Samples	Reads	ASVs	Species	Genera	Families	Orders	Classes	Phyla
Z1	1637	37	32	31	29	19	11	3
Z2	940	25	21	20	18	16	8	2
Z3	20,804	23	20	19	18	12	7	2
L1	8566	24	22	21	20	17	9	2
L2	1575	25	24	22	20	18	11	2
L3	2004	32	31	30	28	22	14	3
Q1	5578	33	33	31	27	21	8	2
Q2	1207	20	18	17	16	15	9	2
Q3	4288	26	25	22	18	14	7	2
CK1	4028	30	29	28	26	22	12	3
CK2	1681	29	31	26	22	18	11	3
CK3	4088	27	29	23	21	18	8	2

**Table A5:** Relative abundances (%) of bacterial phyla in sample groups of different treatments.

Phylum	Z	L	Q	CK	Average
Actinobacteriota	39.17	47.43	65.80	58.79	52.80
Proteobacteria	54.71	42.56	26.20	36.96	40.11
Firmicutes	5.52	9.43	5.12	3.55	5.90
Bacteroidota	0.57	0.57	2.33	0.67	1.03
Bdellovibrionota	0.00	0.00	0.27	0.00	0.07
Patescibacteria	0.00	0.00	0.26	0.00	0.07
Verrucomicrobiota	0.01	0.01	0.01	0.04	0.02
Sumerlaeota	0.02	0.00	0.00	0.00	0.00
Cyanobacteria	0.00	0.00	0.00	0.00	0.00
Acidobacteriota	0.00	0.00	0.00	0.00	0.00
Desulfobacterota	0.00	0.00	0.00	0.0	0.00

**Table A6:** Relative abundances (%) of fungal phyla in sample groups of different treatments.

Phylum	Z	L	Q	CK	Average
Basidiomycota	77.49	74.00	86.13	72.09	77.43
Ascomycota	22.36	25.97	13.87	27.83	22.51
Glomeromycota	0.14	0.00	0.00	0.02	0.04
Zygomycota	0.00	0.03	0.00	0.06	0.02

**Table A7:** Relative abundances (%) of the top 30 dominant bacterial genra in sample groups of different treatments.

Genus	Z	L	Q	CK	Average
*Rhodococcus*	38.70	47.31	64.54	58.30	52.21
*Ralstonia*	23.11	21.27	15.90	14.86	18.78
*Pseudomonas*	12.72	2.00	1.35	0.54	4.15
*Methylobacterium-Methylorubrum*	0.68	1.81	1.25	8.37	3.03
*Rheinheimera*	1.58	2.59	1.11	2.26	1.89
*Lactobacillus*	2.41	1.06	1.70	0.95	1.53
*Acinetobacter*	5.25	0.04	0.03	0.68	1.50
*Staphylococcus*	0.08	5.60	0.01	0.00	1.42
*Acetobacter*	1.45	0.96	1.09	2.15	1.41
*Burkholderia-Caballeronia-Paraburkholderia*	2.66	1.96	0.01	1.02	1.41
*Stenotrophomonas*	1.02	0.68	2.06	0.36	1.03
*Moraxella*	0.00	1.89	0.20	1.32	0.85
*Bradyrhizobium*	1.01	1.73	0.10	0.42	0.82
*Proteiniclasticum*	0.41	0.78	1.14	0.28	0.65
*Vibrio*	0.12	1.02	0.58	0.60	0.58
*Enhydrobacter*	1.77	0.01	0.01	0.01	0.45
*Paucibacter*	0.37	1.07	0.29	0.00	0.43
*Anoxybacillus*	0.16	1.52	0.00	0.00	0.42
*Marvinbryantia*	0.00	0.00	1.66	0.01	0.42
*Hydrogenophilus*	0.19	1.28	0.00	0.00	0.37
*Hydrogenophaga*	0.00	0.00	0.01	1.44	0.36
*Adhaeribacter*	0.00	0.00	1.33	0.00	0.33
*Acetoanaerobium*	0.46	0.00	0.00	0.79	0.31
*Haloimpatiens*	1.11	0.01	0.01	0.01	0.28
*Pseudarcicella*	0.00	0.00	0.95	0.00	0.24
*Comamonas*	0.49	0.38	0.00	0.00	0.22
*Enterococcus*	0.40	0.34	0.08	0.00	0.20
*Peptoniphilus*	0.16	0.00	0.30	0.29	0.19
*Janibacter*	0.00	0.0	0.64	0.00	0.16
*Enterobacter*	0.00	0.00	0.00	0.63	0.16

**Table A8:** Relative abundances (%) of the top 30 dominant fungal genra in sample groups of different treatments.

Genus	Z	L	Q	CK	Average
*Asterotremella*	40.96	36.31	45.03	27.24	37.39
*Malassezia*	22.84	17.89	32.08	18.26	22.77
*Verticillium*	0.00	0.00	0.00	18.34	4.59
*Candida*	0.00	4.89	7.42	3.29	3.90
*Cryptococcus*	0.15	0.00	0.09	14.78	3.76
*Amanita*	0.00	10.60	0.00	0.00	2.65
*Hydnochaete*	8.78	0.30	0.09	0.14	2.33
*Elsinoaceae* *_unclassified*	7.94	0.00	0.00	0.00	1.99
*Pycnoporellus*	0.00	0.00	6.99	0.00	1.75
*Psilocybe*	0.94	0.00	0.23	5.18	1.59
*Hirsutella*	6.10	0.00	0.00	0.00	1.53
*Verrucariaceae_unclassified*	0.00	4.32	0.02	0.00	1.08
*Rigidoporus*	0.12	3.90	0.04	0.00	1.01
*Gibberella*	0.58	1.55	0.83	0.30	0.81
*Perenniporia*	0.00	3.08	0.00	0.16	0.81
*Peniophora*	0.00	0.00	0.00	3.11	0.78
*Pleosporales_unclassified*	2.20	0.27	0.12	0.18	0.69
*Alternaria*	0.42	0.45	0.29	1.15	0.58
*Coniosporium*	0.00	0.00	0.00	1.92	0.48
*Eurotium*	0.00	0.00	1.73	0.12	0.46
*Exobasidium*	0.00	0.00	0.00	1.70	0.43
*Kluyveromyces*	0.00	1.56	0.00	0.00	0.39
*Guignardia*	1.51	0.00	0.00	0.00	0.38
*Sporidiobolus*	1.24	0.00	0.00	0.00	0.31
*Trichosporon*	0.00	0.00	1.13	0.00	0.28
*Herpotrichiellaceae_unclassified*	0.38	0.32	0.16	0.24	0.28
*Nectriaceae_unclassified*	0.00	0.72	0.00	0.32	0.26
*RhodoTorula*	0.12	0.23	0.06	0.57	0.24
*Trichoglossum*	0.25	0.31	0.08	0.24	0.22
*Dothideaceae_unclassified*	0.00	0.00	0.86	0.00	0.22
